# Editorial for the Special Issue: Macro and Microorganism Interactions

**DOI:** 10.3390/microorganisms8111751

**Published:** 2020-11-07

**Authors:** Luciana Giovannetti, Carlo Viti

**Affiliations:** Department of Agriculture, Food, Environment and Forestry (DAGRI), University of Florence, I-50144 Firenze, Italy; luciana.giovannetti@unifi.it

The knowledge of symbiotic, parasitic, and commensal interactions between macro and microorganisms is fundamental to explaining their coexistence, ecology, and productivity. These interactions constitute an extraordinarily complex web that includes trophic structures and molecular communications. The net of interactions between macro and microorganisms is very tight and shows its own metabolic and regulatory processes. Therefore, the terms “holobiont” and “superorganism” were introduced to highlight the complexity of these interactions. Pioneering were the studies of Lynn Margulis, who introduced the concept of “holobiont” into biology in the early 1990s [1]. Nowadays, the use of omics technologies allows for the improvement of our knowledge about many aspects of this emerging and exciting field of research that is an attractor for several scientists.

The present Special Issue, which includes three reviews and seven original manuscripts, covers different aspects of prokaryotic and eukaryotic organism interactions.

In the review from Bulgari et al. [2], the microbe–plant–animal interactions are debated in the frame of the use of microorganisms as biocontrol agents (BCAs); moreover, the risks of the BCA–plant–human pathogen infection cycle are discussed. Pasqua et al., in their review [3], described the role of the multidrug efflux pumps belonging to the major facilitator superfamily (MSF) in the interplay between bacteria and animal host cells. A particular emphasis was put to the role of MSF efflux pumps in bacterial virulence, plant and animal cells colonization and biofilm formation. Tânia Nobre reviewed the literature about the bacteria symbionts of fruit flies, intending to summarize the knowledge on the potential development of a symbiosis-based strategy for olive fruit fly control [4]. Bacteria belonging to *Candidatus* Erwinia dacicola, which are well recognized as obligate endosymbionts of fruit flies, seem to be the most promising target for a symbiotic disruption approach [4].

Out of the seven original manuscripts published, four concerned the bacteria–plant interactions, two the symbiosis between corals and microalgae, and one the microbial community associated with ruminants.

Bradáčová and collaborators investigated the performance of maize inoculated with a microbial consortium of plant-growth promotion microorganisms (PGPMs) [5]. The results obtained demonstrated that the effect of PGPMs on plants was strongly related to the soil features with a significant impact of a high level of organic matter contents combined with stabilized ammonium fertilization. Authors stated that there is a need for better knowledge of the exact conditions required for the induction of beneficial effects on plant growth in PGPM-assisted production strategies.

*Azospirillum* is recognized as plant growth-promoting bacterium, and recently a microbial inoculant containing *Azospirillum* has been used to promote the growth of onions. Nevertheless, there is no information about the relationship between onion and *Azospirillum* strains [6]. In their study, Hong et al. examined the plant cell localization of *A. brasilense* when in *Allium cepa* L. endophytic infection occurs [6]. Moreover, the morphological conversion of *A. brasilense* into cyst-like cells (i.e., c-form) after its initial interaction with onion seedlings was observed.

Zoledowska et al. [7] and Tegli et al. [8] studied the molecular communication in plant–pathogen interactions. The metabolic model of plant–pathogen bacterium *Pectobacterium parmentieri* SCC3139 was reconstructed and, predicting and examining biochemical reactions, permitted the intensive investigation of the nature of communication in plant–microbe interactions [7]. Moreover, the reconstructed metabolic model furnished information about the essential genes to be experimentally tested for bactericide design. Tegli et al. demonstrated that a bacterial MATE transporter was able to mediate IAA efflux in the plant pathogen *Pseudomonas savastanoi* pv. *nerii* strain Psn23, as already known for some plant MATE membrane proteins that mediate transport of several phytohormones, including auxins [8].

Ocean warming has a powerful impact on coral microalgae symbiosis. Meron et al. generated vital apo-symbiotic *Euphyllia paradivisa* corals that lacked the endosymbiotic algae to study the effect of algal presence on coral heat stress response [9]. Heat stress is related to the bleaching events that are a consequence of the disruption of the coral–dinoflagellate symbiosis. De Barros Marangoni et al. focused their activity on peroxynitrite generation and coral bleaching to identify the early warning signs of bleaching [10]. The result obtained showed that peroxynitrite might be an essential mediator of bleaching in scleractinian corals and hydrocorals experiencing heat stress under field conditions.

Buccioni et al. evaluated the effect of addition to diets of chestnut tannins (CHTs) or its single components (e.g., vescalagin or gallic acid) on rumen microbial community [11]. The major effect was found when CHT was added to diets supporting the hypothesis that modulation of rumen community could be obtained when all components are added to diets rather than a single component.

The diverse contributions in this Special Issue showcase progress in our knowledge in macro and microorganism interactions, nevertheless fundamental questions remain unanswered for the intricate network of interactions between the partners.

We hope that the readers enjoy this Special Issue. We want to thank all authors who contributed to this Special Issue as well as the reviewers for precious work during manuscript evaluation.

## References

[1] Margulis L. (1991). Symbiosis as a source of evolutionary innovation: Speciation and morphogenesis. Cambridge MA MLFR; Symbiogenesis and Symbionticism.

[2] Bulgari D., Montagna M., Gobbi E., Faoro F. (2019). Green technology: Bacteria-based approach could lead to unsuspected microbe-plant-animal interactions. Microorganisms.

[3] Pasqua M., Grossi M., Zennaro A., Fanelli G., Micheli G., Barras F., Colonna B., Prosseda G. (2019). The varied role of efflux pumps of the MFS family in the interplay of bacteria with animal and plant cells. Microorganisms.

[4] Nobre T. (2019). Symbiosis in sustainable agriculture: Can olive fruit fly bacterial microbiome be useful in pest management?. Microorganisms.

[5] Bradáčová K., Sittinger M., Tietz K., Neuhäuser B., Kandeler E., Berger N., Ludewig U., Neumann G. (2019). Maize inoculation with microbial consortia: Contrasting effects on rhizosphere activities, nutrient acquisition and early growth in different soils. Microorganisms.

[6] Hong L., Orikasa Y., Sakamoto H., Ohwada T. (2019). Plant tissue localization and morphological conversion of *Azospirillum brasilense* upon initial interaction with *Allium cepa* L.. Microorganisms.

[7] Zoledowska S., Presta L., Fondi M., Decorosi F., Giovannetti L., Mengoni A., Lojkowska E. (2019). Metabolic modeling of *Pectobacterium parmentieri* SCC3193 provides insights into metabolic pathways of plant pathogenic bacteria. Microorganisms.

[8] Tegli S., Bini L., Calamai S., Cerboneschi M., Biancalani C. (2020). A MATE Transporter is Involved in pathogenicity and IAA homeostasis in the hyperplastic plant pathogen *Pseudomonas savastanoi* pv. *nerii*. Microorganisms.

[9] Meron D., Maor-Landaw K., Weizman E., Ben-Asher H.W., Eyal G., Banin E., Loya Y., Levy O. (2019). The algal symbiont modifies the transcriptome of the scleractinian coral *Euphyllia paradivisa* during heat stress. Microorganisms.

[10] de Barros Marangoni L.F., Mies M., Güth A.Z., Banha T.N.S., Inague A., da Silva Fonseca J., Dalmolin C., Coelho Faria S., Ferrier-Pagès C., Bianchini A. (2019). Peroxynitrite generation and increased heterotrophic capacity are linked to the disruption of the coral–dinoflagellate symbiosis in a scleractinian and hydrocoral species. Microorganisms.

[11] Mannelli F., Daghio M., Alves S.P., Bessa R.J.B., Minieri S., Giovannetti L., Conte G., Mele M., Messini A., Rapaccini S. (2019). Effects of chestnut tannin extract, vescalagin and gallic acid on the dimethyl acetals profile and microbial community composition in rumen liquor: An in vitro study. Microorganisms.

